# Rheological Optimization of 3D-Printed Cementitious Materials Using Response Surface Methodology

**DOI:** 10.3390/ma18173933

**Published:** 2025-08-22

**Authors:** Chenfei Wang, Junyin Lian, Yunhui Fang, Guangming Fan, Yixin Yang, Wenkai Huang, Shuqin Shi

**Affiliations:** 1 College of Civil Engineering and Architecture, Xiamen University of Technology, Xiamen 361024, China; 18960027152@163.com (J.L.); 19065495506@163.com (G.F.); imust372658639@163.com (Y.Y.); 17759660249@163.com (W.H.); 15838703894@163.com (S.S.); 2 Engineering Research Center of Structure Crack Control for Major Project, Fujian Province University, Xiamen 361024, China; 3 Xiamen Chengzhi New Materials Technology Co., Ltd., Xiamen 361024, China; 4 College of Civil Engineering, Inner Mongolia University of Science and Technology, Baotou 014010, China; 5 Admixture Research Institute, KZJ New Materials Group Co., Ltd., Xiamen 361011, China; fangyunhui@126.com

**Keywords:** 3D-printed cementitious materials, admixtures, flowability, rheological properties, mixture design approach

## Abstract

This study employed response surface methodology (RSM) to optimize admixture proportions in 3D-printed cementitious materials, with the aim of enhancing printability. Based on preliminary tests, three additives, namely, an accelerator, hydroxypropyl methylcellulose (HPMC), and polycarboxylate superplasticizer (PCE), were incorporated to evaluate their effects on flowability and dynamic yield stress. A Box–Behnken central composite design was used to establish a mathematical model, followed by the RSM-driven optimization of mix proportions. The optimized formulation (0.32% accelerator, 0.24% HPMC, and 0.23% PCE) achieved a flowability of 147.5 mm and a dynamic yield stress of 711 Pa, which closely matched the predicted values and fulfilled the printability requirements, thus establishing RSM as an effective approach for designing printable cementitious composites. This approach established an RSM-based optimization framework for mix proportion design. These findings offer a mechanistic framework for rational 3DPC mixture design, combining theoretical insights and practical implementation in additive construction.

## 1. Introduction

Driven by the advancements in industrial innovation, 3D-printed concrete (3DPC) has emerged as a transformative technology for intelligent and automated construction, marking a paradigm shift in the building sector [1,2,3]. Through digital design and automated processes, 3DPC can facilitate the precise, layer-by-layer deposition of materials based on digital models, thereby constructing complex architectural forms. This approach improves construction efficiency and accelerates timelines, but also minimizes material waste and the environmental footprint [4]. Recent studies have emphasized the rheological properties of 3D-printed cementitious materials, especially flowability and yield stress, as critical indicators of their capacity to resist plastic deformation under shear and retain post-shear fluidity. These metrics offer critical insights into the workability and buildability of cementitious materials, positioning them as key research topics in academia and industry. To address these challenges, researchers such as Panda et al. [5,6,7] engineered cementitious composites with tailored rheological behavior for 3DPC applications. However, current research has often adopted a unidimensional approach, optimizing mix proportions through isolated rheological or buildability parameters, rather than holistically integrating flowability, buildability, and rheology.

Despite their low dosage in material formulations, chemical admixtures play a critical role in enhancing the performance of cementitious systems. Numerous studies have explored how admixtures influence the rheology of 3DPC, with a particular focus on flow behavior and structural stability. For example, Chen et al. [8] enhanced the thixotropy of calcium sulphoaluminate cement paste using thickeners, superplasticizers, and retarders, maintaining a plastic viscosity and dynamic yield stress below 2.5 Pa·s and 645.54 Pa, respectively. This approach optimized the flowability and elevated static yield stress, thus improving the structural stability of printed layers. Chen et al. [8,9,10] investigated the rheological behavior of 3D-printed sulphoaluminate cement composites incorporating cellulose ether, superplasticizers, Li_2_CO_3_, and bentonite. The results revealed that cellulose ether and bentonite significantly increased the static yield stress and plastic viscosity, reducing interlayer deformation. By contrast, superplasticizers and Li_2_CO_3_ were found to negatively influence these rheological parameters. After examining nano-silica and superplasticizers, Kruger et al. [11,12] constructed a rheological–thixotropic model for 3D-printed cementitious materials. The model highlighted how the formation–breakage–reconstruction cycle of flocculation structures governs thixotropic behavior, offering a quantitative basis for assessing printability. Zhu et al. [13] demonstrated that hydroxypropyl methylcellulose (HPMC) markedly improved the rheology of 3D-printed mortar. Furthermore, higher HPMC dosages increased the apparent viscosity, yield stress, and plastic viscosity while inducing a nonmonotonic thixotropic response, initially increasing and then decreasing, which collectively enhanced printability. Therefore, given the inherent complexity of admixture effects on cementitious rheology, systematically elucidating their synergistic or antagonistic interactions is essential for rational 3DPC design.

Despite extensive experimental studies on admixture rheological impacts, systematic investigations into 3DPC remain limited [14]. As a result, developing predictive models via targeted experiments remains critical for optimizing 3DPC printability [5]. Built on the Box–Behnken design, response surface methodology (RSM) provides a framework for investigating the individual and interactive effects of admixtures on rheological properties. RSM offers several benefits including reduced experimental runs, accelerated testing, and a high predictive accuracy. Moreover, its capacity to resolve multi-factor interactions positions RSM as a powerful tool for designing printable cement-based composites. First proposed by Box et al. [15], RSM combines experimental design with mathematical modeling to optimize complex systems. By strategically sampling localized experimental points, RSM can be used to construct regression models to extrapolate global relationships between input variables and output responses to identify optimal parameter combinations [16].

Based on prior single-factor studies by our research team, this work determined the dosage ranges for key experimental factors. A control mixture was supplemented with three chemical admixtures: an accelerator, cellulose ether, and polycarboxylate-based water reducer. Fresh-state rheology and flow behavior (dynamic yield stress) were characterized through standardized rheological tests. The experimental data were analyzed via RSM-driven regression models to quantify the individual and synergistic effects of admixture dosages on the performance metrics. This approach established an RSM-based optimization framework for mix proportion design. These findings offer a mechanistic framework for rational 3DPC mixture design, combining theoretical insights and practical implementation in additive construction.

## 2. Materials and Methods

### 2.1. Raw Materials

**Figure 1:**
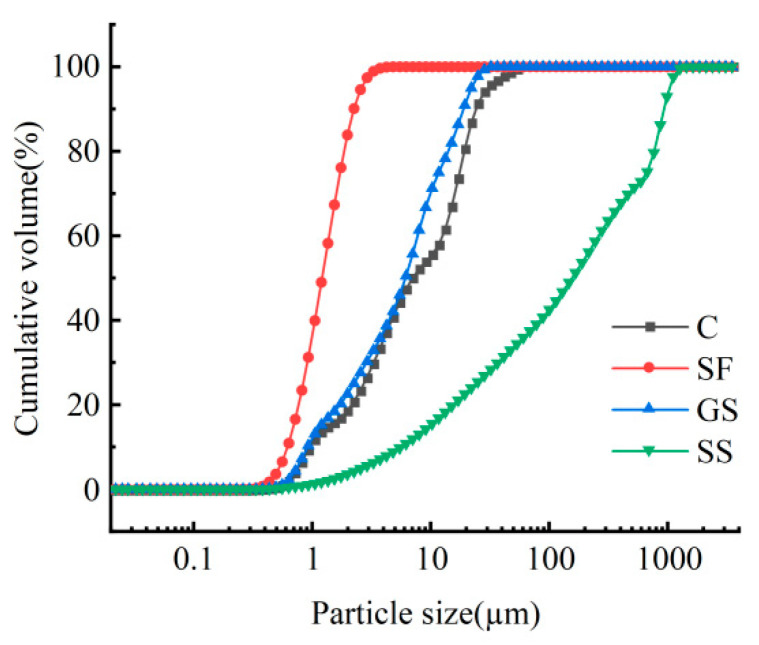
Cumulative particle size distribution curve of the cementitious material and standard sand.

The cement consisted of P·O 42.5 ordinary Portland cement (C), sourced from Huaren Cement (Zhangping, China) Co., Ltd., while the silica fume was produced by Quanzhou Weilinte Import & Export Co., Ltd. (SF), Quanzhou, China. The mineral powder consisted of grade S95 ground granulated blast furnace slag (GS). The detailed chemical compositions are listed in Table 1. Particle size distribution analysis was performed using a Bettersize 3000Plus laser particle size analyzer (Fangxu Technology (Shanghai) Co., Ltd., Shanghai, China), with the cumulative particle size distribution curves shown in Figure 1. The sand consisted of natural sand (SS), with a particle size of 0.5–1 mm, and 6 mm polypropylene fibers (PPFs) were used, with their main properties shown in Table 2. The admixture contained a Kezhijie alkali-free accelerating agent that was used to extend the printable time of the mortar; hydroxypropyl methylcellulose (HPMC) ether solution that was used to enhance the printable properties of the mortar; and Kezhijie polycarboxylic acid water reducer (SO7D), with a water reduction rate of 40%. Polycarboxylate superplasticizer was used to adjust the workability of the fresh mortar. Tap water was used in the experiments.

According to the preliminary test, the content of the basic mix ratio could be determined as follows. The water–binder ratio was 0.3, with a cement/silica fume/mineral powder ratio of 1:0.3:0.15, and cement/sand/ratio of 1.45:1.5, with 2% PPF content of the cementitious material volume. Based on the closely packed theory combined with mixing experience, when the cement volume content accounts for 60–70% of the total powder, and the silica fume/cement and mineral powder/cement ratios are within the ranges of 0.2–0.3 and 0.15–0.3, respectively, the concrete material can achieve the maximum density [17]. The water-to-binder ratio and fiber content significantly influence the strength of fiber-reinforced concrete, with the water-to-binder ratio being the key factor. A higher fiber content increases concrete toughness but reduces workability [18]. In existing studies, fiber content is generally selected to be around 2% of the concrete volume [19].

### 2.2. Test Methods

#### 2.2.1. Rheological Test

**Figure 2:**
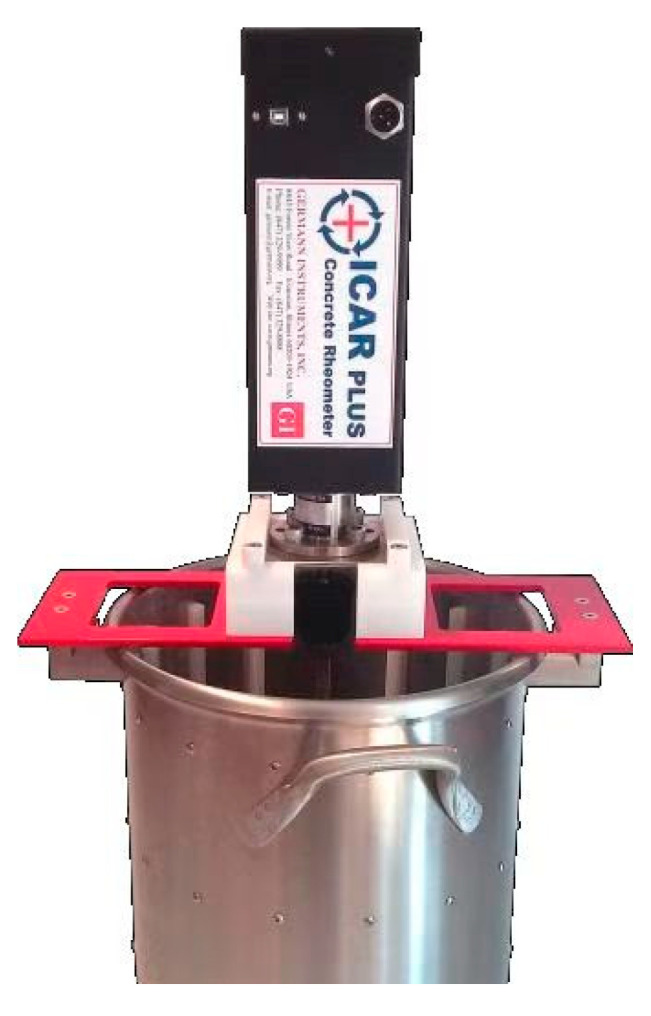
ICAR Plus concrete rheometer.

In this study, the ICAR Plus concrete rheometer was used to determine the rheological properties of fresh concrete. The rheometer results are shown in Figure 2, where the blade radius is 63.5 mm, the blade height is 127 mm, and the container radius is 143 mm. The test procedure is as follows: The test protocol involved rheological characterization, which was performed using an ICAR Plus concrete rheometer. Pre-shearing was conducted at 0.5 rotations per second (rps) for 20 s, and (2) linear ramp-down was performed from 0.5 to 0.05 rps to complete shear profiling. Each group was measured three times and the average result was calculated.

The rheological behavior of the material followed the Bingham model, and the yield stress (Pa) and plastic viscosity (Pa·s) of fresh concrete were determined by fitting the experimental data from its rotational speed–torque relationship. The dynamic yield stress was calculated using Equation (1), while the static yield stress was derived from Equation (2) as follows:

(1) $$\tau =\tau_{0}+\mu \gamma ,$$

(2) $$\tau_{s}=\frac{2T}{\pi D^{3}(\frac{H}{D}+\frac{1}{3})},$$

where $\tau_{0}$ is the yield stress, *T* is the maximum torque, *D* is the blade diameter, *H* is the blade height, $\tau_{s}$ denotes the static yield stress, μ is the plastic viscosity, and γ is the shear rate.

#### 2.2.2. Flowability Test

The flowability testing protocol (compliant with GB/T 2419-2005 [20]) was conducted as follows. To prepare the samples, fresh mortar was poured into a truncated conical mold, compacted layer-wise, and vertically demolded after consolidation. For flow measurement, the flow table was triggered to release 25 drops in 25 s. Post test, the spread diameter was measured twice orthogonally, and the mean value was recorded. Each group was measured three times and the average result was calculated.

#### 2.2.3. Preparation Procedures

**Figure 3:**
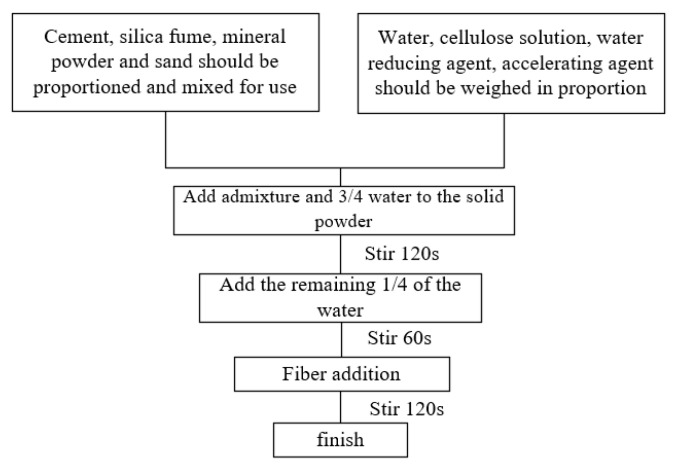
Three-dimensional printing material preparation flow chart.

The preparation procedure for the 3D-printable materials is illustrated in Figure 3, with the detailed workflow is outlined below.

(1) Dry mixing was achieved by mixing the pre-weighed cement, silica fume, slag powder, and sand in a planetary mixer for 180 s to homogenize the binder components.
(2) The chemical admixtures and 75% of the total water were then slowly introduced into the dry mixture and wet-mixed for 120 s, to ensure even dispersion.
(3) Water adjustment was then performed, where the remaining 25% of water was incrementally added during a 60 s mixing cycle to optimize the rheology and batch consistency.
(4) Fibers were then integrated by manually feeding the fibers into the matrix and blending for 120 s to ensure uniform distribution, after which mixing was stopped to minimize fiber breakage.

### 2.3. Test Program

#### 2.3.1. Response Surface Method Test Design

Despite their minor dosage (<5% by mass), chemical admixtures govern key performance metrics in 3D-printed mortars, which require strict rheological control and structural buildability [21,22]. For successful printing, these materials must demonstrate exceptional flowability under pumping shear, transitioning rapidly to high early strength after extrusion. Consequently, admixtures must fulfill a dual functionality: shear-thinning behavior for extrusion and rapid stiffening for dimensional stability.

Yield stress and flowability serve as critical parameters for assessing the resistance of 3DPC to shear-induced plastic deformation and their recovery of post-shear fluidity. These parameters critically govern a material’s workability and buildability, offering fundamental insights into its thixotropic behavior [23,24]. This study adopted a single-factor design to quantify the effects of accelerators, cellulose ether, and superplasticizers on the rheology of 3DPC, establishing a rational framework for formulation design and quality control.

##### Single-Factor Experiment

(1) Effect of the accelerator on rheological properties

The base mix components were batched by mass. With PCE fixed at 0.25% and HPMC at 0.15%, the accelerator dosages (0.2–0.6%) were incrementally varied to evaluate their effects on rheological properties.

(2) Effect of HPMC on the rheological properties

The mix constituents were prepared according to standardized protocols. The accelerator and PCE were fixed at 0.2% and 0.25%, respectively, and the HPMC dosages (0.11–0.27%) were adjusted to assess their influence on rheological behavior.

(3) Effect of PCE on the rheological properties

The base mixture was prepared using standardized protocols. The accelerator and HPMC were maintained at 0.2% and 0.15%, while the PCE dosages (0.2–0.4%) were modulated to quantify their impact on rheology.

##### The Experimental Design of Response Surface Optimization

Building on the baseline mix design and single-factor experiments, three independent variables were selected, namely, the accelerator (A), HPMC (B), and PCE (C). Dynamic yield stress and flowability were assigned as response metrics, and a Box–Behnken design-based RSM was implemented using Design Expert 13 software [24]. Factor levels (Table 3) were calibrated according to the single-factor test outcomes (Section 2.3.1), ensuring alignment with practical dosage ranges.

Post-processing optimization analysis, supplemented by validation tests, was performed to determine the optimal 3DPC mix formulation. A quadratic regression model (Equation (3)) was subsequently adopted to correlate the input factors (accelerator, HPMC, and PCE) and output responses (dynamic yield stress and flowability) as follows:

(3) $$Y=\beta_{0}+\sum_{i=1}^{k}\beta_{i}X_{i}+{\sum_{i=1}^{k}\beta_{\mathrm{ii}}X_{i}}^{2}+\sum_{i<j}^{k}\beta_{\mathrm{ij}}X_{i}X_{j}$$

where Y is the response variable, $\beta_{0}$ denotes the model intercept, $\beta_{i}$ represents the linear effect of variable $X_{i}$, $\beta_{ii}$ denotes the quadratic effect of $X_{i},{\;\beta }_{ij}$ characterizes the interaction effect between $X_{i}$ and $X_{j}$, and $X_{i}$ and $X_{j}$ are independent variables.

The predictive accuracy of the response surface model was evaluated via the coefficient of determination (R^2^) and adjusted determination coefficient ($R_{adj}^{2}$), as demonstrated in Equations (4) and (5).

(4) $$R^{2}=\frac{\sum_{i=1}^{k}({\overset{\wedge }{y}}_{i}-{\bar{y}}_{i}{)}^{2}}{\sum_{i=1}^{k}(y_{i}-{\bar{y}}_{i}{)}^{2}}$$

(5) $${R^{2}}_{\mathrm{adj}}=1-\frac{\sum_{i=1}^{k}(y_{i}-{\bar{y}}_{i}{)}^{2}(k-1)}{\sum_{i=1}^{k}(y_{i}-{\bar{y}}_{i}{)}^{2}(k-f-1)}$$

where k is the number of experimental runs, f represents the degrees of freedom, $y_{i}$ is the observed response value, ${\overset{\wedge }{y}}_{i}$ denotes the predicted response value, and ${\bar{y}}_{i}$ is the mean observed response value. Model accuracy was deemed satisfactory when both R^2^ and $R_{adj}^{2}$ exceeded 0.9, with values approaching 1 indicating superior fitting precision.

#### 2.3.2. Three-Dimensional Printing Experiment Design

##### 3D Printer Parameter Settings

This experiment utilized a concrete 3D printer (Xiamen Zhichuangchi Technology Co., Ltd., Key, Xiamen, China), with the operational parameters of the 3D printer configured as follows: nozzle diameter of 20 mm, X-/Y-axis movement speed of 80 mm/s for the print head, and Z-axis movement speed of 40 mm/s.

##### Print Path Diagram

**Figure 4:**
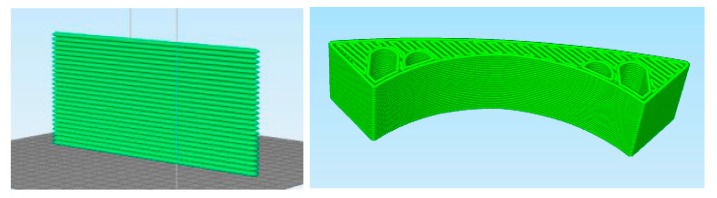
Model slice diagram.

Buildability, essential for successful 3D printing, requires printed layers to resist structural collapse and significant deformation under self-weight and overburden loads from successive layers. Deformation resistance under gravitational forces, quantified by the form retention ratio, directly reflects material stability and serves as a critical metric for assessing stackability in 3DPC. Higher form retention ratios are typically correlated with reduced self-weight deformation, ensuring stable multi-layer deposition. The buildability assessment protocol in this work followed established methodologies, utilizing a predefined printing path configuration (Figure 4) to simulate sequential layer deposition under controlled conditions. This framework enabled the systematic evaluation of interlayer adhesion and time-dependent structural integrity.

## 3. Results and Discussion

### 3.1. Single-Factor Test Results

The effect of additive dosage on the rheological properties of the material was investigated through single-factor experiments. This investigation had two primary objectives, with the first exploring the extent to which different variables could affect the rheological parameters of the material, and the second providing guidance for subsequent formulation optimization. These objectives were achieved by analyzing the influence of different variables on the rheological properties of the material.

(1) Effect of accelerator on the rheological properties

**Figure 5:**
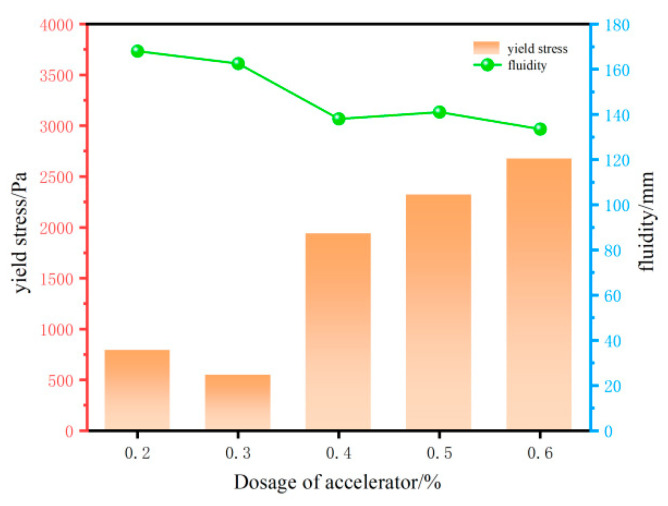
Effect of accelerant on the rheological properties of 3DPC.

As shown in Figure 5, under fixed experimental conditions, the dynamic yield stress of 3DPC exhibited a complex dependence on accelerator dosage (0.2–0.6%). Below 0.3%, the yield stress decreased sharply, likely due to the insufficient modification of hydration kinetics, which delayed the formation of interparticle bonds and weakened early structural networks. Beyond 0.3%, the accelerated nucleation of calcium–silicate–hydrate (C-S-H) gels dominated, resulting in a progressive yield stress increase [25]. Concurrently, flowability decreased across the dosage range, except for a transient increase at 0.5% attributed to localized saturation effects. Based on these trends, accelerator dosages of 0.3%, 0.4%, and 0.5% were identified as critical thresholds for further optimization.

(2) Effect of HPMC solution on the rheological properties

**Figure 6:**
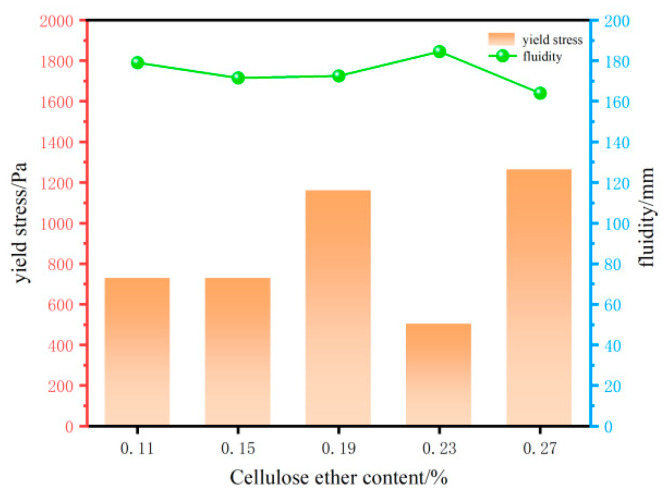
Effect of cellulose ether on rheological properties of 3DPC.

As shown in Figure 6, under identical conditions, the yield stress and flowability of the 3D-printed cement-based materials exhibited significant changes when the hydroxypropyl methylcellulose ether solution content ranged from 0.11% to 0.27%, with notable variations observed at 0.23% and 0.27%. This phenomenon could be attributed to the propensity of the hydroxyl groups and ether bonds present on the polymer chains of the HPMC solution, which readily formed hydrogen bonds with water. This process resulted in a reduction in free water content within the paste, thereby increasing internal friction within the material. Consequently, this heightened internal friction led to an increase in yield stress [8,26], and concurrently, the incorporation of HPMC markedly diminished the flowability of the mortar. As the HPMC content increased from 0.11% to 0.27%, the initial flowability of the mortar experienced a decrease from 179 mm to 171.5, 172.5, 184.5, and 164 mm. As a high-molecular-weight polymer, HPMC contained molecules that could intertwine, thereby forming a network structure. This structure enhanced the cohesive strength of the cement paste by enveloping components such as Ca(OH)_2_, resulting in the improved cohesiveness of the mortar at the macro level. As the static time increased, the hydration degree of the mortar increased, leading to a time-dependent loss of flowability [27,28,29]. Notably, at a content level of 0.23%, the mortar achieved its maximum flowability retention capacity. A series of optimization experiments were conducted, taking into account all pertinent factors. The experiments incorporated HPMC content levels of 0.19%, 0.23%, and 0.27%.

1. Effect of PCE on rheological properties

**Figure 7:**
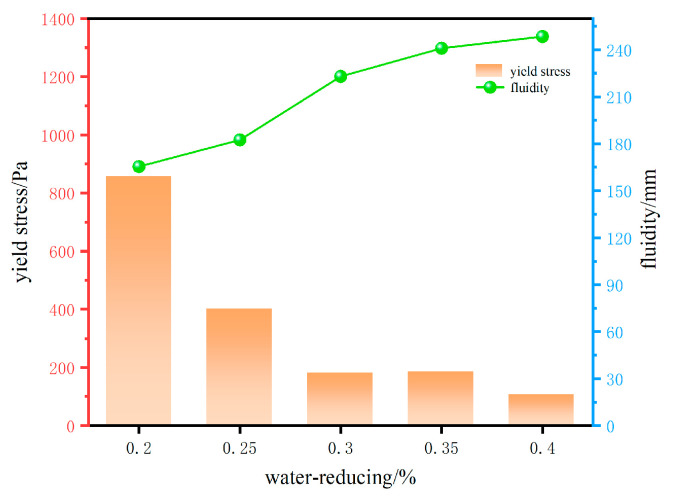
Effect of water-reducing agent on the rheological properties of 3DPC.

As illustrated in Figure 7, under identical conditions, the yield stress of the 3D-printed cement-based materials gradually decreased with increasing PCE dosage in the range of 0.2–0.4%, while the flowability correspondingly increased. This phenomenon could be attributed to the addition of high-performance superplasticizers to the cement paste, which resulted in changes to the paste’s internal structure. The adsorption of high-performance water-reducing agents on the surface of the cement particles was found to alter the zeta potential of the particles [30]. This change also possibly resulted in steric hindrance, leading to a reduction in the cohesive forces between the particles and an increase in repulsive forces. The flocculation structure of the cement paste underwent significant disruption, thus enhancing the dispersion of cement particles and establishing a novel equilibrium system. This, in turn, resulted in changes to the internal structure of the paste, which enhanced its flowability. In addition to relying on electrostatic repulsive forces, the polycarboxylic acid high-performance water-reducing agent also possessed a comb-shaped branched structure that could exert steric hindrance effects in the cement paste, promoting a more thorough dispersion of the cement [31,32,33]. To assess printability, optimization experiments were conducted using concentrations of 0.2%, 0.25%, and 0.3%.

### 3.2. Response Surface Results

The experimental configurations and outcomes of the RSM-based study are detailed in Table 4, including the design parameters and optimization results.

#### 3.2.1. Response Surface Regression Model Analysis

Guided by the Box–Behnken design methodology, a predictive regression model was developed to establish the response equations for systematic optimization.

The experimental results in Table 4 were analyzed, with factors A (accelerator), B (hydroxypropyl methylcellulose, HPMC), and C (polycarboxylate superplasticizer, PCE) denoting the three admixtures under investigation. Interaction terms AB, AC, and BC quantified the synergistic or antagonistic relationships between the admixtures, modulating the key rheological responses.

**Figure 8:**
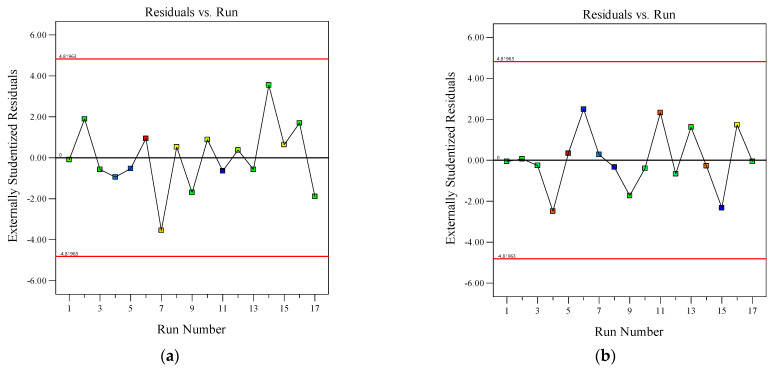
Relationship between the residuals and the run order for flowability (**a**) and the dynamic yield stress (**b**) tests.

Figure 8 illustrates the relationship between the residuals, the run order for flowability (a), and the dynamic yield stress (b) tests, indicating no significant trends or clustering patterns. Residuals were randomly dispersed around the central line, confirming their mutual independence. From a statistical perspective, the derived RSM model was rigorously justified. It should be noted that the experimental points in the figure are divided into two categories: red points are high impact/outliers marked by the software, and the rest are normal data. Occasional red points do not need to be removed. If the diagnostic indicators and overall model quality are still acceptable, they can be retained and used for reference only.

**Figure 9:**
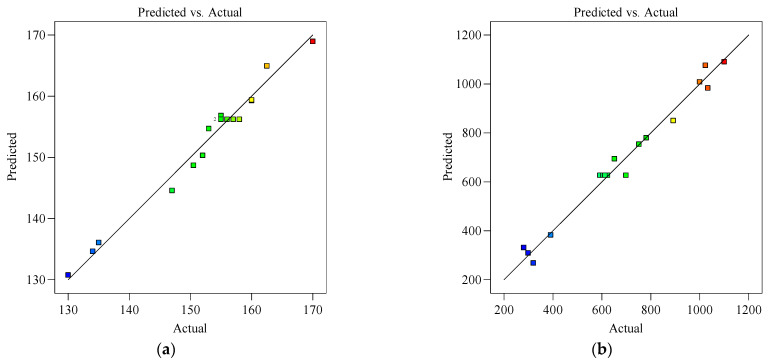
Predicted and actual material properties: (**a**) flowability; (**b**) dynamic yield stress.

Figure 9a compares the predicted flowability (calculated via the response surface model) with the experimental values, while Figure 9b illustrates the predicted rheological properties (derived from the response surface model) against their experimentally measured counterparts. The linear regression line (y(predicted) = x(actual)) indicated a high degree of agreement. Flowability and dynamic yield stress both exhibited goodness-of-fit metrics (R^2^ and $R_{adj}^{2}$) exceeding 0.9, fully satisfying the model accuracy requirements, which confirmed that the proposed model effectively captured the relationship between the material characteristics and compositional parameters.

The final empirical models correlating flowability and dynamic yield stress to the admixture dosages (A, B, C) and their interactions were derived as follows:

Flowability = 156.20 − 3.12A − 0.9375B + 13.31C + 0.125AB + 1.13AC + 1.00BC + 2.02A^2^ − 5.60B^2^ − 4.60C^2^ (6)

Dynamic Yield Stress = 626.40 + 45.58A − 32.47B − 358.30C − 2.65AB + 11.75AC + 21.35BC + 73.97A^2^ + 68.82B^2^ − 16.77C^2^. (7)

**Figure 10:**
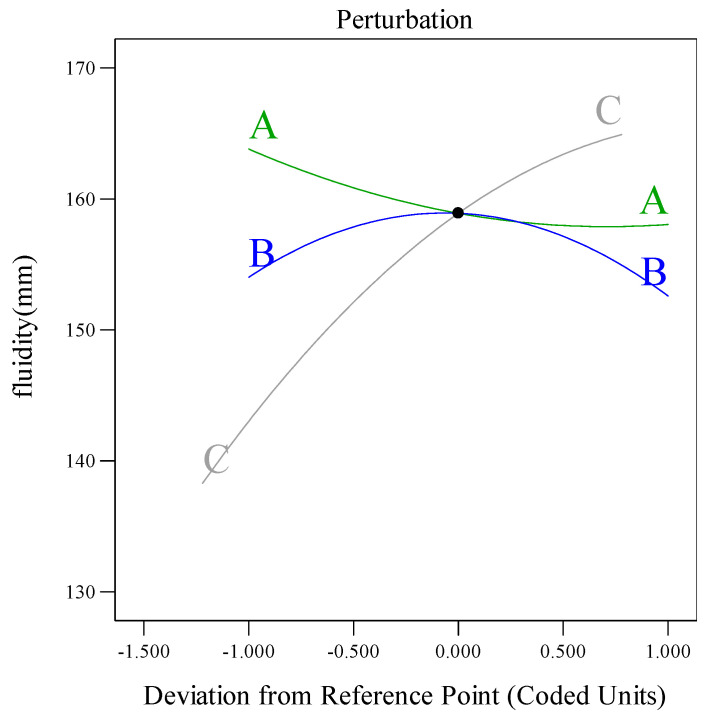
Influence of different components on flowability.

**Figure 11:**
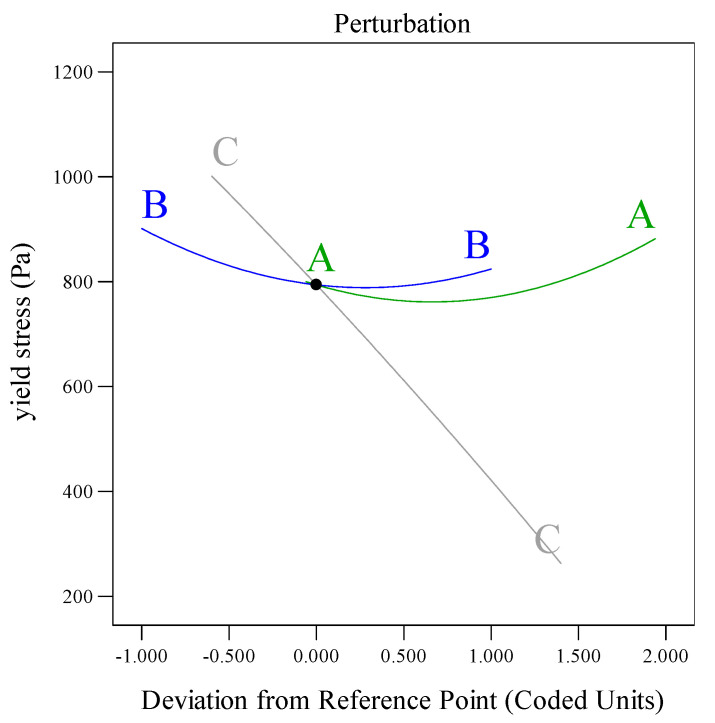
Effects of different components on the dynamic yield stress.

As shown in Table 5 and Table 6, the flowability model (*p* < 0.0001) and dynamic yield stress model (*p* < 0.0001) exhibited significant validity (*p* < 0.05). The lack-of-fit values (0.0656 and 0.1770) exceeded 0.05, confirming strong agreement between the models and experimental data [34,35]. The F-values indicated that the factor influence hierarchy followed C > A > B for both flowability and dynamic yield stress. In the flowability model, A, C, and BC interactions (*p* < 0.05) significantly governed flow behavior, while the AB interaction showed a negligible impact. For dynamic yield stress, only factor C (*p* < 0.05) demonstrated significant dominance, with interactions involving A and B remaining statistically insignificant. The sensitivity analysis of trend curves (Figure 10 and Figure 11) quantified the model responsiveness to dosage variations across the components. The results showed that flowability decreased linearly with A, indicating a parabolic trend with B, and rising sharply with C. Meanwhile, the dynamic yield stress declined with B or C but gradually increased with A, with C exhibiting the strongest destabilizing effect.

#### 3.2.2. Response Surface Analysis

**Figure 12:**
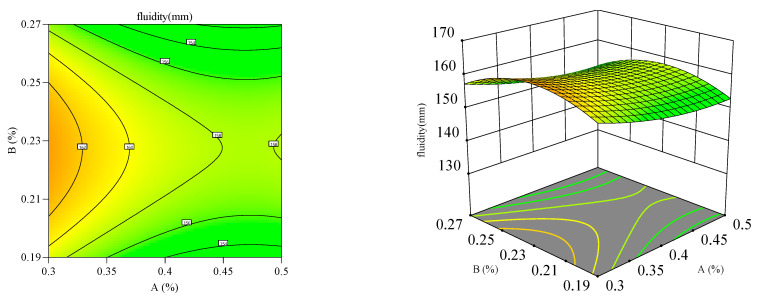
Effect of accelerant and HPMC on the flowability of the mortar.

**Figure 13:**
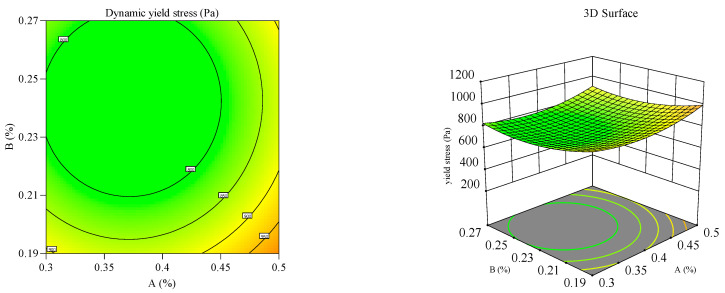
Effect of accelerant and HPMC on the yield stress of the mortar.

**Figure 14:**
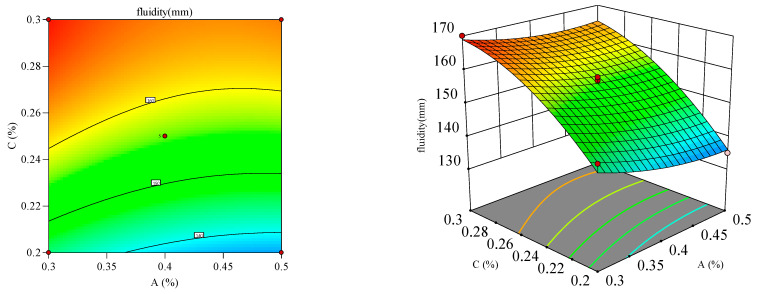
Effect of accelerant and PCE on the flowability of the mortar.

**Figure 15:**
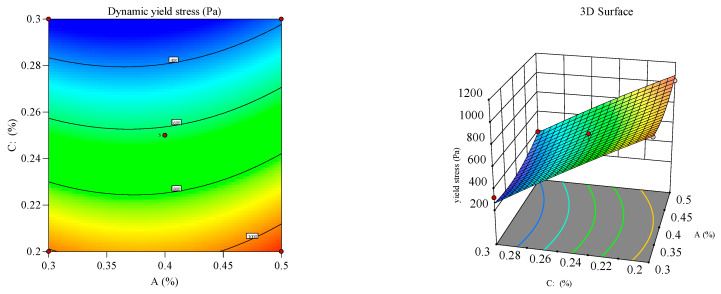
Effect of accelerant and PCE on the yield stress of the mortar.

**Figure 16:**
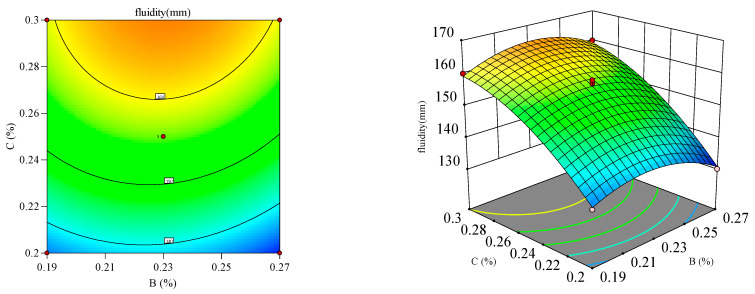
Effect of HPMC and PCE on the flowability of the mortar.

**Figure 17:**
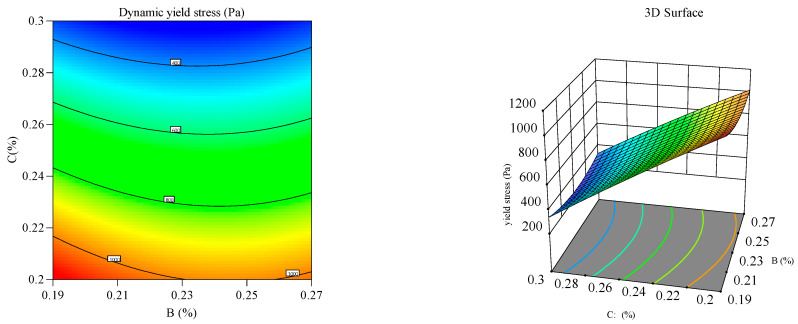
Effect of HPMC and PCE on the yield stress of the mortar.

Response surface and contour plots (Figure 12, Figure 13, Figure 14, Figure 15, Figure 16 and Figure 17) were generated using Design Expert 13 to analyze the multi-factor interactions between A, B, and C on the flowability and yield stress, guided by ANOVA-validated regression models [36,37,38]. Subsequently, three-dimensional response surfaces and contour plots were derived by fixing one factor at its central value, thus isolating the interaction effects of the remaining two variables on flowability and yield stress. Inter-factor interactions were quantified using surface curvature and contour density, where steeper slopes and tighter contours denoted pronounced nonlinear dependencies. Elliptical contours, a hallmark of strong synergies, highlighted cooperative interactions between the variables, aligning with prior studies [39,40]. It is important to note that in the generated 3D response surface plot, colors only indicate the high/low or strong/weak response values.

Figure 12, Figure 13, Figure 14, Figure 15, Figure 16 and Figure 17 illustrate the interactive effects of A, B, and C on the mortar flowability and dynamic yield stress. The A–B interaction at fixed C levels (Figure 12 and Figure 13) revealed competing effects, notably, flowability declined and dynamic yield stress increased with increasing A near the central B dosages [41,42]. At moderate B doses, a higher A content decreased flowability but promoted the dynamic yield stress. This behavior arose from aluminum sulfate (A) reacting vigorously with the cement phases (C3S/C3A), accelerating hydration kinetics, particle coagulation, and early structuration at the expense of workability. At elevated A dosages, flowability showed a parabolic response to B, initially rising then declining, due to the saturation of hydroxyl-mediated hydration suppression [43]. In addition, B enhanced flow retention through hydrogen-bonded water entrainment and polymeric film formation, inhibiting moisture evaporation and sustaining workability [44].

Figure 14 and Figure 15 depict the mutual influence of A and C on flowability and dynamic yield stress at fixed B levels, where flowability escalated linearly with a higher C dosage, whereas dynamic yield stress declined proportionally. Mechanistically, the C polymeric side chains adsorbed onto cement particles, forming hydration films that stabilized the colloidal system through dual steric–electrostatic repulsion, thus suppressing particle agglomeration. The resultant dispersion enhanced flowability by reducing interparticle friction, but destabilized the cohesive forces, thereby lowering dynamic yield stress [45].

Figure 16 and Figure 17 depict the synergistic interaction between B and C on flowability at fixed A levels. A higher B dosage amplified flowability gains with increasing C, driven by complementary dispersion and water retention mechanisms. The steep response surface curvature (*p* < 0.05) confirmed significant B–C interactions, which was validated by ANOVA. Meanwhile, dominant B–C synergy emerged from the adsorption of B onto cement surfaces, which synergistically amplified superplasticizer dispersion.

### 3.3. Response Surface Results Optimization and Verification

Rheological parameters serve as key indicators for assessing the deformation behavior and workability of cementitious pastes, with direct implications for predicting flow dynamics in 3D-printed systems [46,47]. Well established thresholds [10,48,49] for 3DPC include (1) static yield stress (900–2189 Pa); (2) dynamic yield stress (200–800 Pa); (3) plastic viscosity (<25 Pa·s).

Extrudability, a prerequisite for 3D printing, demands uninterrupted mortar flow through the nozzle to prevent clogging. Extrudability relies on flowability, with inadequate flowability compromising pumpability and extrusion stability. Considering printer heterogeneity, the flowability range of printable 3DPC materials can be empirically established as 150–200 mm [13,50,51].

**Figure 18:**
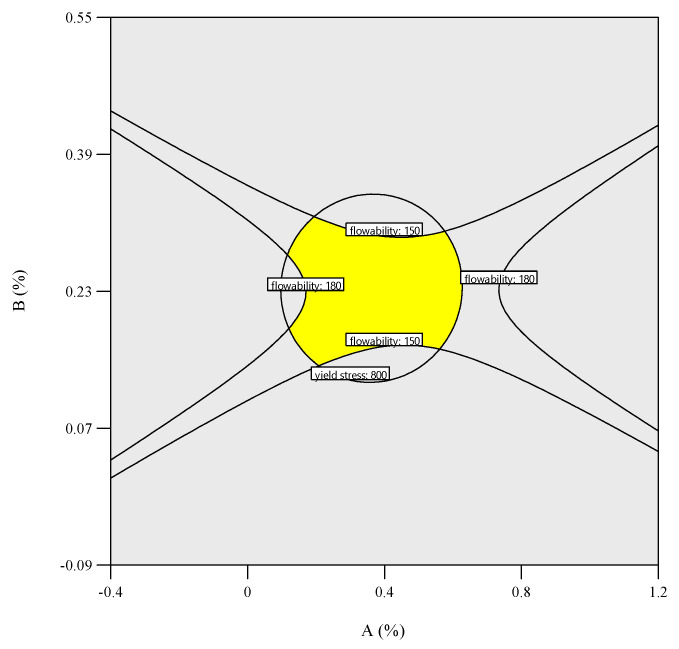
Double response overlay plot of flowability and dynamic yield stress.

**Figure 19:**
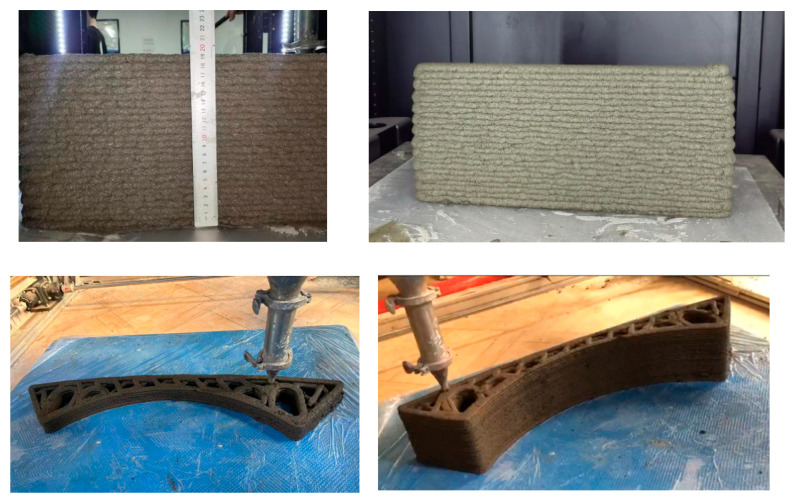
Performance validation plot for optimized mix ratio in 3D printing tests.

Flowability (150–180 mm) and dynamic yield stress (200–800 Pa), critical for extrudability and buildability, were designated as dual-response criteria for 3DPC mix optimization. An overlay plot (Figure 18) was used to integrate the flowability and yield stress models (Section 2.3.2) to identify feasible parameter combinations, using the multi-response optimization module in Design Expert 13. The intersecting feasible region (yellow) represented admixture dosages meeting both the flowability and yield stress thresholds. The desirability function (0 < D < 1) was used to rank feasible solutions, maximizing congruence with target responses. The optimal proportions (Table 7) predicted the flowability (153.8 mm) and yield stress (768.0 Pa) within 5% of the experimental data. Experimental validation (A: 0.32%, B: 0.24%, C: 0.23%) achieved 147.5 mm flowability (4.1% deviation) and 711 Pa yield stress (7.6% deviation), which aligned with the model predictions (<5% deviation). Buildability tests (Figure 19) confirmed stable interlayer adhesion and dimensional coherence under cumulative loading, satisfying the 3D printing requirements. Additional mechanical tests show that the material achieves a 3-day strength of up to 21.4MPa, a 7-day strength of up to 30.5 MPa, and a 28-day strength of up to 40.6 MPa, with a printable time of 40 min.

## 4. Conclusions

### 4.1. Main Conclusions

Building on a baseline mix design, a Box–Behnken central composite design was adopted in this study to conduct response surface experiments for optimizing mortar formulation. Subsequently, a quadratic regression model with excellent goodness-of-fit was established. Compared with traditional orthogonal experimental methods, the response surface methodology (RSM) offered a clearer visualization of factor interactions through 3D surface plots, aiding in identifying synergistic effects. The optimal mix ratio resulted in a mortar flowability and a dynamic yield stress which closely aligned with the RSM predictions, demonstrating the feasibility of the optimization framework. The following conclusions were drawn:

(1) Model validity was established, where a statistical analysis of the quadratic polynomials for flowability and dynamic yield stress confirmed a high goodness-of-fit (R^2^ > 0.95) and robust predictive accuracy across responses.
(2) Factor significance
(3) Flowability followed the order of polycarboxylate superplasticizer (PCE) > accelerator > hydroxypropyl methylcellulose (HPMC) solution, with significant interaction between HPMC and PCE. Dynamic yield stress followed the order of PCE > accelerator > HPMC solution.
(4) The optimal mix consisted of accelerator dosage = 0.32%, HPMC solution = 0.24%, and PCE dosage = 0.23%. The experimental results showed 147.5 mm flowability (4.1% deviation) and 711 Pa dynamic yield stress (7.6% deviation), aligning closely with the predictions.

The minimal deviation between the predicted and measured values demonstrated the efficacy of RSM in optimizing 3D printing material formulations. After applying the RSM model, building material production can be accurately proportioned in one stage, reducing waste and costs. Three-dimensional printing gains a stable and adjustable flow window and real-time equipment linkage, enabling efficient, reliable, and low-waste additive construction.

### 4.2. Suggestions and Prospects

This study investigates the optimization of the mixture proportions for 3D-printed cementitious materials using response surface methodology. Nevertheless, several challenges remain and further research is needed in the following areas:

(1) This study only uses flowability and dynamic yield stress to judge printability, but it does not consider other important factors like thixotropy, how well layers stick together, or how long the material stays workable (open time). Focusing on just two factors may miss the other key points needed for successful 3D printing.
(2) The printing tests used only one optimized mix and did not try different shapes or printing conditions. Testing more types of prints would make the results stronger and more widely useful.
(3) All tests were performed in a laboratory environment using limited material quantities, and the RSM model was based on some simplifying assumptions. Therefore, the applicability of the findings in actual 3D printing or construction projects requires further validation through field-based continuous printing and long-term structural performance monitoring.

## Figures and Tables

**Table 1:** Chemical components of the cement, silica powder, and mineral powder (% by mass).

	CaO	SiO_2_	Al_2_O_3_	Fe_2_O_3_	MgO	SO_3_	K_2_O	Na_2_O	Loss
C	64.00	20.10	5.55	2.55	2.37	3.86	0.66	0.25	3.50
SF	1.05	94.91	0.78	0.45	0.56	0.57	0.66	0.50	0.52
GS	37.92	31.38	13.77	9.82	1.09	0.07	1.52	0.33	0.23

**Table 2:** Main properties of the polypropylene fibers.

	Length (mm)	Diameter (mm)	Young’s Modulus (GPa)	Elastic Modulus (GPa)	Tensile Strength (MPa)
PPF	6	0.02	13.2	3-5	>480

**Table 3:** Design factor level (% by mass).

Factor	Level		
	−1	0	1
A	0.3	0.4	0.5
B	0.19	0.23	0.27
C	0.2	0.25	0.3

**Table 4:** Response surface test design and results (% by mass).

No.	A Accelerator (%)	B HPMC (%)	C PCE (%)	Flowability (mm)	Dynamic Yield Stress (Pa)
1	0.3	0.19	0.25	155	751.6
2	0.5	0.19	0.25	152	765.7
3	0.3	0.27	0.25	153	651.6
4	0.5	0.27	0.25	150.5	662.2
5	0.3	0.23	0.2	147	999.9
6	0.5	0.23	0.2	135	950.6
7	0.3	0.23	0.3	170	320.2
8	0.5	0.23	0.3	162.5	355
9	0.4	0.19	0.2	134	1183.3
10	0.4	0.27	0.2	130	1111.6
11	0.4	0.19	0.3	160	233.9
12	0.4	0.27	0.3	160	200
13	0.4	0.23	0.25	157	592.1
14	0.4	0.23	0.25	155	698.5
15	0.4	0.23	0.25	156	623.2
16	0.4	0.23	0.25	158	605.4
17	0.4	0.23	0.25	155	612.8

**Table 5:** Regression model analysis of variance (flowability).

Source	Sum of Squares	df	Mean Square	F-Value	*p*-Value	
Model	1754.27	9	194.92	38.86	<0.0001	significant
A	78.12	1	78.12	15.57	0.0056	
B	7.03	1	7.03	1.40	0.2751	
C	1417.78	1	1417.78	282.65	<0.0001	
AB	0.0625	1	0.0625	0.0125	0.9143	
AC	5.06	1	5.06	1.01	0.3485	
BC	4.00	1	4.00	0.7974	0.0415	
A^2^	17.27	1	17.27	3.44	0.1059	
B^2^	132.04	1	132.04	26.32	0.0014	
C^2^	89.09	1	89.09	17.76	0.0040	
Residual	35.11	7	5.02			
Lack of Fit	28.31	3	9.44	5.55	0.0656	not significant
Pure Error	6.80	4	1.70			
Cor Total	1789.38	16				
R^2^					0.9804	
R^2^_Adj_					0.9551	
R^2^_pred_					0.7409	

**Table 6:** Regression model analysis of variance (dynamic yield stress).

Source	Sum of Squares	df	Mean Square	F-Value	*p*-Value	
Model	1.100 × 10^6^	9	1.222 × 10^5^	39.90	<0.0001	Significant
A	16,616.65	1	16,616.65	5.42	0.0527	
B	8437.00	1	8437.00	2.75	0.1410	
C	1.027 × 10^6^	1	1.027 × 10^6^	335.25	<0.0001	
AB	28.09	1	28.09	0.0092	0.9264	
AC	552.25	1	552.25	0.1803	0.6839	
BC	1823.29	1	1823.29	0.5952	0.4657	
A2	23,041.27	1	23,041.27	7.52	0.0288	
B2	19,944.76	1	19,944.76	6.51	0.0380	
C2	1184.84	1	1184.84	0.3868	0.5537	
Residual	21,444.47	7	3063.50			
Lack of Fit	14,433.37	3	4811.12	2.74	0.1770	Not significant
Pure Error	7011.10	4	1752.77			
Cor Total	1.122 × 10^6^	16				
R2					0.9596	
R2Adj					0.9076	
R2pred					0.8522	

**Table 7:** Response surface optimization optimal mix ratio.

Accelerator	HPMC	PCE	Flowability (mm)	Dynamic Yield Stress (Pa)	Desirability
0.321%	0.241%	0.231%	153.763	768.031	1

## Data Availability

The original contributions presented in this study are included in the article. Further inquiries can be directed to the corresponding author.
